# Supplementary figures and images for: Synthesis of Full-Length cDNA Infectious Clones of Soybean Mosaic Virus and Functional Identification of a Key Amino Acid in the Silencing Suppressor Hc-Pro
Source: Viruses. 2020 Aug 13;12(8):886. doi: 10.3390/v12080886 (PMC7472419; doi:10.3390/v12080886)

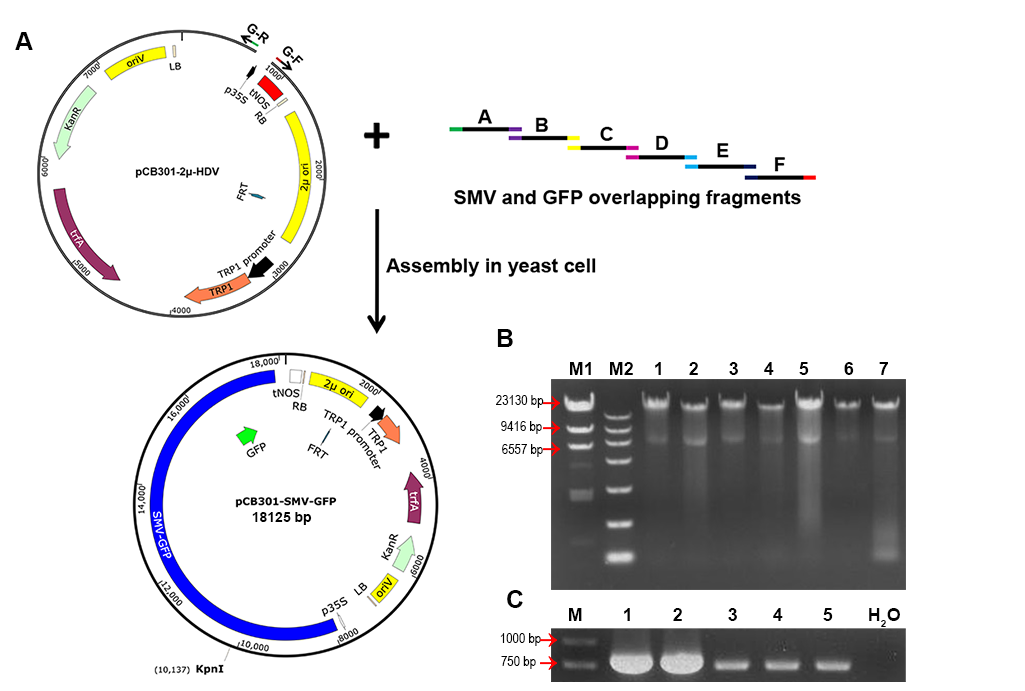

Supplement: Supplementary file 1 [file viruses-12-00886-s001.zip › S2.tif]

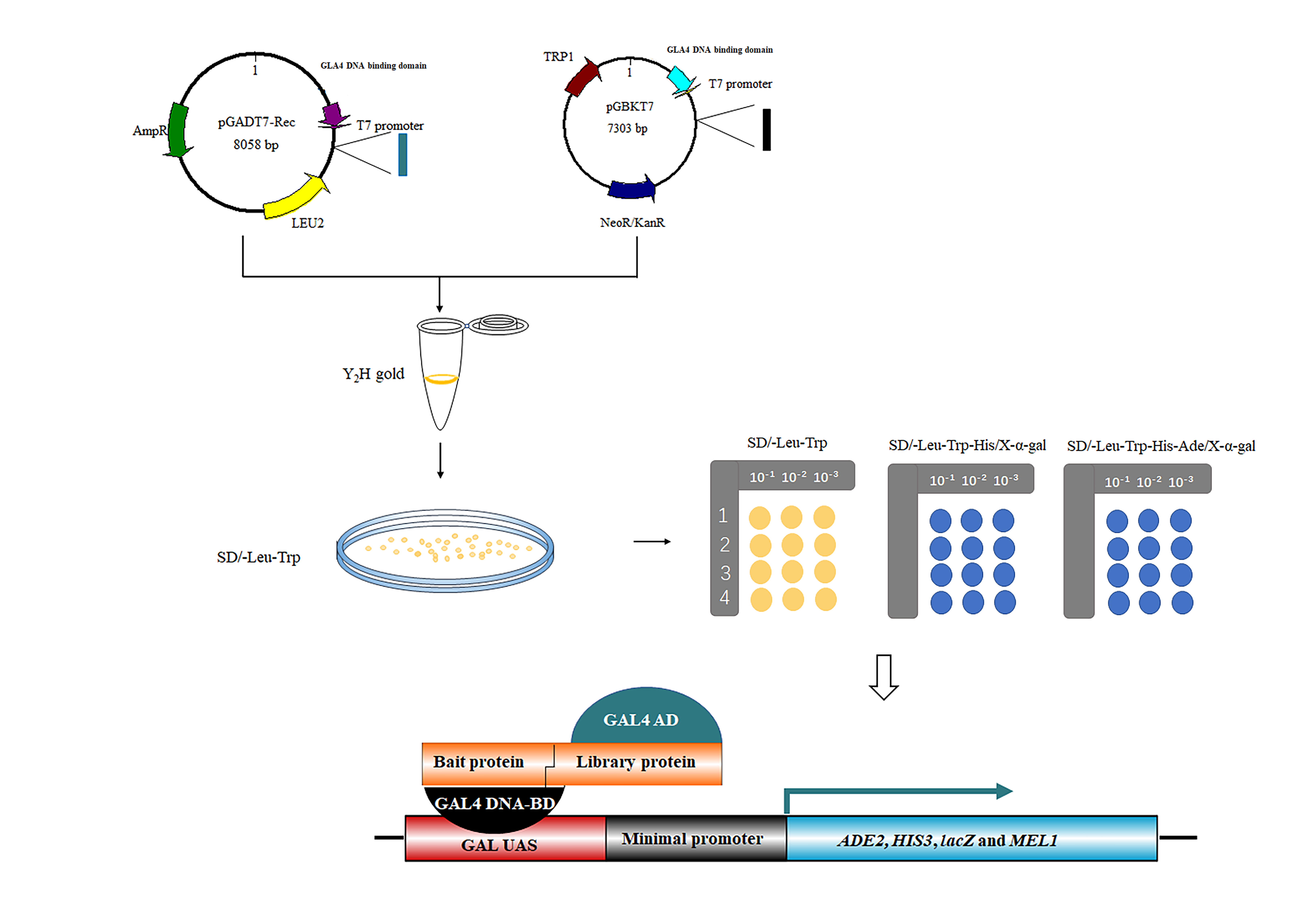

Supplement: Supplementary file 1 [file viruses-12-00886-s001.zip › S3.tif]

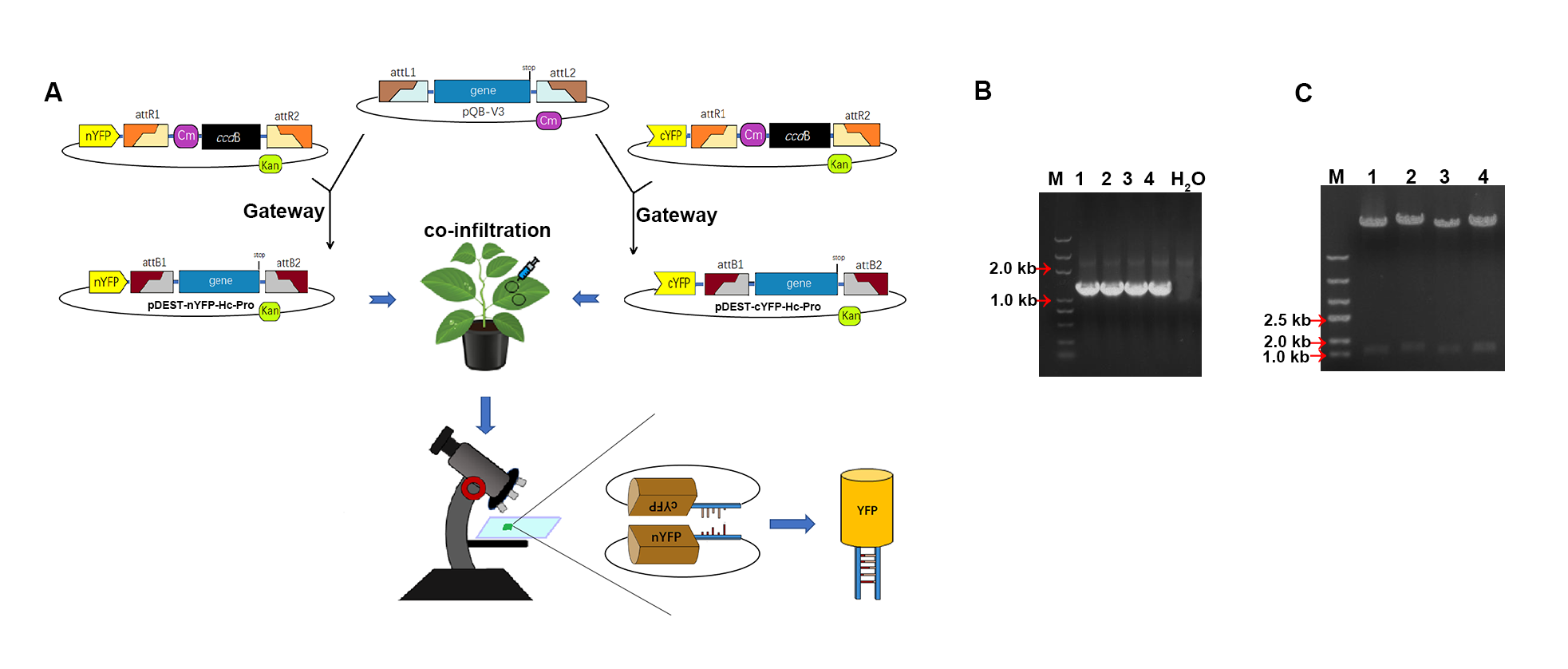

Supplement: Supplementary file 1 [file viruses-12-00886-s001.zip › S4.tif]

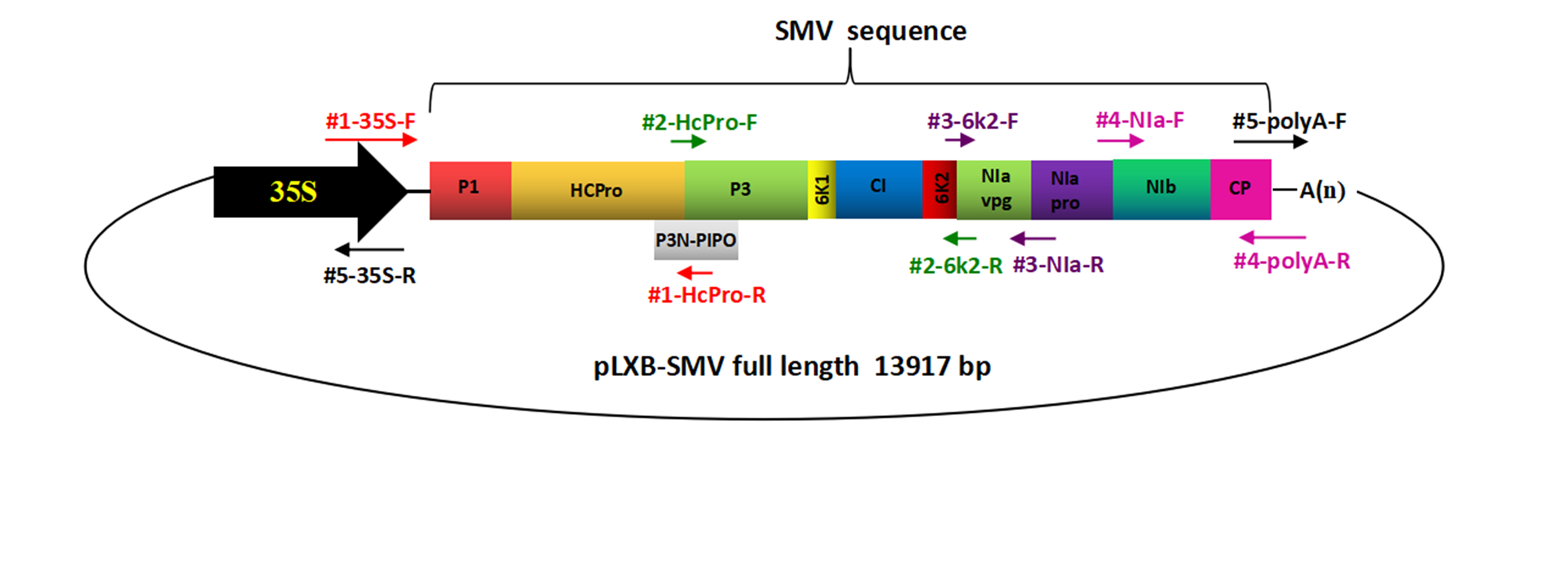

Supplement: Supplementary file 1 [file viruses-12-00886-s001.zip › S1.tif]
